# Predictors of Telehealth Use Among Cancer Survivors: Retrospective Study

**DOI:** 10.2196/87403

**Published:** 2026-07-03

**Authors:** Jasskiran Kaur, Jeffrey Zhong, Carley Mitchell, Annie Zhang, Qian Wang, Melinda L Hsu

**Affiliations:** 1 University Hospitals Seidman Cancer Center, 11100 Euclid Avenue, Cleveland, OH, 44106, United States, 1 216-286-6505

**Keywords:** survivorship, telehealth, digital health, internet, disparities, healthcare delivery, cancer

## Abstract

**Background:**

As the number of cancer survivors continues to grow, optimizing long-term survivorship care models has become increasingly important. Telehealth has the potential to improve access to health care for survivors; however, studies evaluating telehealth in this population remain limited. Additionally, concerns persist regarding equity in technology access and digital literacy.

**Objective:**

This study aimed to examine demographic factors and patient attitudes influencing telehealth use among cancer survivors compared to the general population.

**Methods:**

Adult participants were identified from the nationally representative database Health Information National Trends Survey 6 (HINTS 6). Multivariable logistic regression was used to calculate the predictors of telehealth use among cancer survivors. *χ*^2^ tests compared the prevalence of reported reasons of not using telehealth in the last 12 months between cancer survivors and the general population.

**Results:**

A total of 5793 (weighted n=239,557,883) individuals were included in this study, 7.7% (weighted n=18,545,434) who are cancer survivors. 5092 individuals from the general population and 701 cancer survivors were included. Older age was associated with lower telehealth use (adjusted odds ratio [aOR] 0.11; 95% CI 0.02‐0.59 for patients aged ≥65, compared to those under 40 y old). Higher education (aOR 2.55; 95% CI 1.24‐5.27) and heart disease history (aOR 2.52; 95% CI 1.20‐5.28) were associated with increased telehealth use. Employed (aOR 0.46; 95% CI 0.22‐0.97) and retired (aOR=0.37; 95% CI: 0.18‐0.77) cancer survivors were less likely to use telehealth than unemployed individuals. Of the nonusers, over 60% reported that telehealth options were not offered, and 80% preferred in-person visits. Technical issues and privacy concerns were not major factors in utilizing telehealth.

**Conclusions:**

Despite greater telehealth use among cancer survivors, a negative association between older age and telemedicine utilization persists. Efforts should focus on improving access for older cancer survivors and addressing employment-related factors, patient attitudes, and telehealth availability. Future studies should explore personalized approaches to enhance cancer survivors’ health care experiences. Our findings emphasize the need to address specific factors including age and employment related disparities, patient preferences and telehealth availability to optimize equitable access to telehealth and enhance the delivery of cancer survivorship care.

## Introduction

Cancer survivors represent approximately 5.4% of the US population, and the number of survivors is expected to increase by 7.9 million by 2040 [1]. As the number of cancer survivors grows, the need to optimize long-term survivorship care models is imperative. Cancer survivors face unique health challenges, as they require continued monitoring for risk of recurrence, assessment for long-term physical and psychosocial effects of both the disease and its treatments, management of comorbidities, screening for new malignancies, and health promotion. Moreover, cancer survivors may face considerable economic burden for myriad reasons, including the inability to work and the rising costs of cancer therapies [2-4]. Patients with cancer and cancer survivors represented a vulnerable population during the rise of the COVID-19 pandemic, and fear associated with contracting the virus significantly affected all aspects of medical care [5-7]. As a result, telehealth offered potential health care delivery solutions for all socially distanced patients [8].

Telehealth is defined as the delivery of health and health-related services via telecommunication technologies [9] and has been successfully implemented over the last two decades in noncancer contexts including diabetes care, hypertension, mental health, and chronic pain [10-13]. Telehealth for cancer patients was evaluated before the COVID-19 pandemic and was shown to be as effective at improving quality of life as in-person care [14]. Although prior studies have examined telehealth use among cancer survivors, these have largely been limited to specific cancer types [15,16]. Data on telehealth utilization among cancer survivors, particularly relative to the general population, remain limited. In our analyses, the general population serves as the reference group, enabling assessment of whether cancer survivorship is independently associated with the outcomes of interest.

While telehealth is a powerful tool with the potential to improve health care outcomes, inequities remain regarding access to the technologies required for successful telehealth interactions. This is exemplified by a decrease in telehealth utilization by adults who are uninsured, have lower incomes, and are without high school degrees [17]. Older adults may have less digital literacy as well, and a small qualitative study found older cancer survivors made use of online platforms for general health concerns but preferred in-person visits for major issues [18,19]. Further research regarding the use of and potential barriers to telehealth services is imperative to continued improvement of survivorship-care. Our study aims to analyze demographic factors and patient attitudes that influence telehealth uptake among cancer survivors compared to the general population.

## Methods

This study was a retrospective cross-sectional analysis of publicly available data from the Health Information National Trends Survey 6 (HINTS 6) [20]. HINTS data were assessed from the National Cancer Institute HINTS website. which collects nationally representative data about how adults use different communication channels to obtain health information. Telehealth visits were defined in HINTS 6 as a telephone or video appointment. Telehealth data was extracted for adult patients from March 7, 2022, to November 8, 2022. A total of 5793 individuals were included in this study, 5092 individuals from the general population and 701 cancer survivors. Our outcome of interest was overall telehealth utilization rates, reasons for having telehealth visits, and patient perceptions of telehealth utilization.

### Outcome Variables

Participants were first asked whether they had used telehealth (defined as a telephone or video visit with a health care provider) in the past 12 months and whether they had been offered a telehealth option for care. Telehealth was assessed using items from section D of HINTS 6 [20].

Among those who used telehealth, additional questions assessed reasons for choosing telehealth (eg, provider recommendation, convenience, infection avoidance, or ability to include caregivers). Participants were also asked about their experiences and perceptions of telehealth, including preferences for in-person versus telehealth visits, concerns about privacy, perceived difficulty using the technology, technical problems encountered, and whether the quality of care was comparable to in-person visits.

Among those who did not use telehealth, items assessed whether telehealth was offered and reasons for nonuse (eg, preferred in person, privacy concerns, perceived difficulty using the technology)

Statistical analysis included a *χ*^2^ test to compare the prevalence of telehealth use in the last 12 months between cancer survivors and the general population. Additionally, multivariable logistic regression was used to calculate the adjusted odds ratio (aOR) of telehealth use in cancer survivors compared to the general population. Our model was adjusted for multiple variables — age, sex, race/ethnicity income, education, marital status, BMI, employment status, insurance status, physical activity, diabetes, hypertension, lung disease, heart disease and smoking status.

All analyses incorporated the HINTS complex survey weights, and variance estimates were calculated using the Taylor series linearization method to account for the survey’s stratified sampling design. Listwise deletions were used to treat missing values across these variables. All statistical analyses were performed with SAS 9.4. A 2-sided *P* value of <.05 was considered statistically significant.

### Ethical Considerations

HINTS is deidentified, publicly available data, and has been designated as exempt research and approved by the Westat IRB [21].

## Results

### Patient Characteristics

A total of 239,557,883 individuals were included in the weighted sample, of which 7.7% (weighted n=18,545,434) were cancer survivors. 5092 individuals from the general population and 701 cancer survivors were included. Cancer survivors differed from the general population across multiple sociodemographic and clinical characteristics (Table 1). As expected, cancer survivors were more likely to be older (*P*<.001) and retired (*P*<.001). Cancer survivors were also more likely than the general population to be female (*P*<.001), non-Hispanic White (*P*<.001), married (*P*=.01), and have insurance (*P*<.001). Clinically, cancer survivors were more likely to be smokers (*P*<.001) and have additional medical comorbidities including diabetes (*P*=.002), hypertension (*P*<.001), heart disease (*P*<.001), and lung disease (=.003).

**Table 1.** Table 1. Demographic features comparing general population vs noncancer survivors 2022 (Weighted N=239,557,883)^a^.

Variables	General population n=5092 (weighted n=221,012,449); 92.3%		Cancer survivors n=701 (weighted n=18,545,434); 7.7%		*P* value
	Unweighted n	Weighted % (95% CI)	Unweighted n	Weighted % (95% CI)	
Age					<.001
18‐39	1213	35.2 (32.6‐37.7)	17	3.6 (1.3‐5.8)	
40‐64	2202	45.5 (43.1‐48.0)	215	39.8 (33.6‐46.0)	
65+	1677	19.3 (17.9‐20.7)	469	56.7 (50.3‐63.0)	
Sex					<.001
Female	3068	49.9 (47.2‐52.6)	437	61.8 (56.6‐66.9)	
Male	2024	50.1 (47.4‐52.8)	264	38.2 (33.1‐43.4)	
Race					<.001
NHW^b^	2731	60.1 (57.4‐62.8)	450	77.1 (72.3‐81.9)	
NHB^c^	791	11.3 (10.2‐12.5)	90	7.4 (5.3‐9.5)	
Hispanic	917	17.5 (15.4‐19.5)	68	9.0 (5.7‐12.2)	
Others	429	11.1 (8.6‐13.6)	39	6.6 (3.7‐9.4)	
Education					.53
≤ High school	1245	28.4 (26.4‐30.5)	180	26.2 (22.0‐30.5)	
Some college	1446	38.9 (36.3‐41.5)	212	42.1 (37.1‐47.0)	
≥ College	2385	32.7 (30.5‐34.9)	304	31.7 (28.0‐35.4)	
Income (USD)					.78
≤50,000	2210	37.4 (35.1‐39.8)	322	36.7 (31.8‐41.5)	
>50,000	2876	62.6 (60.2‐64.9)	376	63.3 (58.5‐68.2)	
Marital status					.01
Unmarried	2442	44.7 (42.4‐47.1)	352	36.9 (31.5‐42.4)	
Married	2626	55.3 (52.9‐57.6)	344	63.1 (57.6‐68.5)	
Employment status					<.001
Unemployed	1212	27.1 (24.0‐30.1)	142	21.5 (16.3‐26.7)	
Employed	2556	56.2 (53.4‐59.0)	180	30.2 (23.7‐36.8)	
Retired	1317	16.7 (15.4‐18.0)	374	48.3 (41.9‐54.7)	
Insurance status					<.001
No	446	11.0 (9.3‐12.8)	13	2.8 (0.5‐5.1)	
Yes	4612	89.0 (87.2‐90.7)	687	97.2 (94.9‐99.5)	
Smoking status					<.001
Never	3293	66.8 (64.7‐68.8)	373	50.9 (45.1‐56.8)	
Current	554	12.0 (10.4‐13.6)	73	12.4 (8.4‐16.3)	
Former	1187	21.2 (19.6‐22.8)	245	36.7 (30.9‐42.5)	
BMI					.62
Underweight/normal	1519	31.4 (29.2‐33.5)	218	30.1 (24.9‐35.2)	
Overweight	1664	33.6 (31.1‐36.2)	241	36.5 (31.5‐41.5)	
Obese	1810	35.0 (32.7‐37.2)	229	33.4 (28.3‐38.6)	
Moderate intensivity activity (min/wk)					.06
≤60	2210	43.0 (40.8‐45.2)	326	51.6 (45.3‐57.9)	
60‐150	1125	21.2 (19.2‐23.1)	139	19.9 (15.2‐24.6)	
151‐300	1006	21.7 (18.9‐24.5)	134	17.3 (13.0‐21.7)	
>300	644	14.2 (12.3‐16.1)	79	11.1 (8.0‐14.3)	
Diabetes	1050	16.6 (15.0‐18.3)	198	25.8 (20.8‐30.8)	.001
Hypertension	2194	36.0 (33.7‐38.2)	428	55.5 (51.0‐59.9)	<.001
Heart disease	462	7.0 (6.0‐7.9)	120	16.3 (12.4‐20.1)	<.001
Lung disease	658	11.4 (10.1‐12.7)	141	19.6 (14.7‐24.4)	.003
Depression	1674	35.6 (33.4‐37.9)	214	32.7 (27.6‐37.8)	.32
Cancer information					
Type of cancer					—^d^
Liquid			38	4.0 (2.2‐5.8)	
Solid			469	67.8 (62.8‐72.8)	
Others			186	28.3 (23.2‐33.3)	
Time since diagnosis					—^d^
≤5 y			272	34.6 (29.5‐39.7)	
>5 y			587	65.4 (60.3‐70.5)	

a Values are presented as unweighted n with weighted percentages and 95% confidence intervals. Percentages and confidence intervals were calculated using survey weights and therefore may not correspond directly to the unweighted counts.

b NHW: Non-Hispanic White.

c NHB: Non-Hispanic Black.

d P-values are not reported because these variables were collected exclusively in the cancer cohort and were not compared between groups.

Telehealth use was more common among cancer survivors than the general population (n=320, 48.2% vs n=2042, 38.4%; *P*=.01, aOR 1.48; 95% CI 1.07‐2.04) (Table 2). Among cancer survivors who utilized telehealth, older age was associated with a lower likelihood of using telehealth compared to those under 40 years old (aOR 0.11; 95% CI 0.02‐0.59 for age ≥65). Additionally, telehealth usage among cancer survivors was greater among those with higher education levels (aOR 2.55; 95% CI 1.24‐5.27) and a history of heart disease (aOR 2.52; 95% CI 1.20‐5.28). However, cancer survivors who were employed (aOR 0.46; 95% CI 0.22‐0.97) or retired (aOR 0.37; 95% CI 0.18‐0.77) were less likely to use telehealth than those who were unemployed (Table 3).

**Table 2.** Comparing telehealth use and perception of telehealth use, comparing cancer survivors to the general population.

Telehealth utilization	General population n=5092 (weighted n=221,012,449); 92.3%		Cancer survivors n=701 (weighted n=18,545,434); 7.7%		*P* value	aOR^a^ 95% CI^b^
	Unweighted n	Weighted % (95% CI)	Unweighted n	Weighted % (95% CI)		
Overall						
Had telehealth	2042	38.4 (36.2‐40.5)	320	48.2 (41.4‐54.9)	.01	1.48 (1.07‐2.04)
Among individuals who used telehealth						
Reason for having televisit						
Recommended by provider	1364	72.9 (70.0‐75.7)	224	79.4 (73.3‐85.4)	.07	1.51 (0.97‐2.35)
Avoid infection	947	51.5 (47.5‐55.5)	114	36.9 (27.6‐46.2)	.008	0.63 (0.40‐0.996)
Convenience	1242	66.6 (63.1‐70.0)	158	56.6 (47.2‐66.1)	.05	0.80 (0.50‐1.30)
Including friends/families	378	22.6 (19.1‐26.2)	68	23.5 (15.3‐31.8)	.84	0.99 (0.55‐1.76)
Having technical difficulty in telehealth visit	380	18.2 (14.7‐21.7)	80	25.7 (18.2‐33.1)	.08	1.36 (0.82‐2.25)
Think telehealth is as good as in person	1439	75.8 (72.0‐79.6)	214	75.3 (69.3‐81.3)	.90	0.90 (0.60‐1.33)
Has privacy concerns with telehealth	285	15.9 (11.7‐20.1)	40	10.4 (6.1‐14.7)	.07	0.43 (0.21‐0.89)

a aOR: adjusted odds ratio.

b Adjusted for age, sex, race, income, education, marital status, BMI, employment status, insurance status, physical activity, diabetes, hypertension, lung disease, heart disease and smoking status.

**Table 3.** Predictors of receiving telehealth.

Variable	Using telehealth
	aOR^a^ 95% CI
Cancer survivor	
No	Ref
Yes	1.48 (1.07‐2.04)^b^
Among cancer survivors only - predicting factors^c^	
Age	
18‐39	Ref
40‐64	0.18 (0.03‐1.03)
65+	0.11 (0.02‐0.59)
Gender	
Female	Ref
Male	1.54 (0.87‐2.71)
Race	
NHW^d^	Ref
NHB^e^	0.75 (0.33‐1.70)
Hispanic	1.77 (0.70‐4.50)
Others	0.88 (0.35‐2.25)
Education	
≤ High school	Ref
Some college	1.93 (0.98‐3.82)
≥ College	2.55 (1.24‐5.27)
Income (USD)	
≤50,000	Ref
>50,000	0.96 (0.53‐1.74)
Marital status	
Unmarried	Ref
Married	0.90 (0.48‐1.70)
Employment status	
Unemployed	Ref
Employed	0.46 (0.22‐0.97)
Retired	0.37 (0.18‐0.77)
Insurance status	
No	Ref
Yes	1.52 (0.30‐7.60)
Smoking status	
Never	Ref
Current	0.62 (0.24‐1.61)
Former	1.40 (0.80‐2.44)
BMI	
Underweight/normal	Ref
Overweight	0.72 (0.38‐1.36)
Obese	1.06 (0.50‐2.23)
Moderate intensity activity (min/wk)	
≤60	Ref
60‐150	1.43 (0.68‐3.00)
151‐300	0.72 (0.35‐1.49)
>300	1.58 (0.69‐3.63)
Diabetes	
No	Ref
Yes	0.89 (0.51‐1.55)
Hypertension	
No	Ref
Yes	1.35 (0.77‐2.35)
Heart disease	
No	Ref
Yes	2.52 (1.20‐5.28)
Lung disease	
No	Ref
Yes	1.57 (0.86‐2.88)
Cancer information	
Type of cancer	
Solid	Ref
Liquid	1.07 (0.34‐3.35)
Others	0.82 (0.44‐1.54)
Time since diagnosis (years)	
≤5	Ref
>5	0.95 (0.52‐1.75)

a aOR: adjusted odds ratio.

b Model adjusted for cancer survivor status, age, sex, race/ethnicity, income, education, marital status, BMI, employment status, insurance status, physical activity, diabetes, hypertension, lung disease, heart disease and smoking status.

c Model adjusted for age, sex, race/ethnicity, income, education, marital status, BMI, employment status, insurance status, physical activity, diabetes, hypertension, lung disease, heart disease, smoking status, type of cancer, and time since cancer diagnosis.

d NHW: Non-Hispanic White.

e NHB: Non-Hispanic Black.

Among those utilizing telehealth, avoiding infection was a more frequent motivator for the general population compared to cancer survivors (n=947, 51.5% vs n=114, 36.9%; *P*=.008, aOR 0.63; 95% CI 0.40‐0.996). No significant differences were observed between cancer survivors and the general population in other reasons for telehealth use, including provider recommendation, convenience, or the ability to include family and friends. Similarly, there were no significant differences in perceptions of telehealth, including perceived difficulties, or equivalence to in-person visits (Table 2). Although privacy concerns were significantly less likely among cancer survivors after adjustment for covariates (aOR 0.43; 95% CI 0.21‐0.89) (Table 2), the unadjusted comparison did not reach statistical significance (*P*=.07).

Among individuals who had not used telehealth in the past 12 months, over 80% (general population, n =411 and cancer survivors, n=68) did not use telehealth because they preferred in-person visits, and over 60% (general population, n=1875 and cancer survivors, n=231) of both groups reported that their health care providers did not offer telehealth services (Table 4). There were no significant differences in privacy concerns or technical difficulties as reasons for not utilizing telehealth (Table 2).

**Table 4.** Among those who did not have telehealth last year, exploring reasons—comparing cancer survivors versus the general population.

Variables	General population n=5092 (weighted n=221,012,449); 92.3%		Cancer survivors n=701 (weighted n=18,545,434); 7.7%		*P *value	aOR^a^ 95% CI^b^
	Unweighted n	Weighted % (95% CI)	Unweighted n	Weighted % (95% CI)		
Offered by providers					.02	
Yes	474	16.6 (14.1‐19.1)	76	20.6 (14.4‐26.7)		1.60 (1.02‐2.49)
No	1875	62.9 (59.6‐66.1)	231	67.6 (60.9‐74.3)		1.24 (0.87‐1.78)
Did not schedule appointment	532	20.5 (17.5‐23.5)	41	11.8 (6.7‐16.9)		0.82 (0.45‐1.48)
Prefer in person	411	83.7 (73.8‐93.5)	68	88.9 (78.1‐99.6)	.48	0.35 (0.06‐2.02)
Privacy concerns	71	17.1 (7.4‐26.7)	26	14.9 (3.8‐26.0)	.76	1.41 (0.39‐5.07)
Technical difficulty	95	17.8 (12.3‐23.3)	16	22.1 (10.6‐33.6)	.51	0.81 (0.30‐2.17)

a aOR: adjusted odds ratio.

b Using the general population as the reference group, model was adjusted for age, sex, race, income, education, marital status, BMI, employment status, insurance status, physical activity, diabetes, hypertension, lung disease, heart disease and smoking status.

## Discussion

### Principal Results

Our study retrospectively analyzed telehealth utilization in both the general population and cancer survivors, using the HINTS 6 data. We identified several factors that may affect the likelihood of cancer survivors utilizing telehealth.

We found older age and lower education level in cancer survivors to be associated with decreased use of telehealth, which is consistent with results from prior research [17-19,22-24]. Chen et al [25] found that prostate cancer survivors with less than a high school education had lower use of telehealth than those who had received any college education. Moreover, there is evidence that cancer patients with higher education levels have more positive perceptions of telemedicine with regards to satisfaction and privacy [26]. Along these same lines, Kjeldsted et al [27] noted that cancer patients with lower education levels reported less positive experiences with telemedicine, specifically with regard to comfort and confidence levels in teleconsultations.

Cancer survivors in our study with a history of heart disease were more likely to use telehealth. Inglist et al [28] published a Cochrane systematic review identifying 41 randomized controlled trials and found telehealth interventions reduced the relative risk of all-cause mortality by 20%. Consequently, various telehealth interventions have been used in heart disease management including structured telephone or video support for monitoring risk factors monitoring such as hypertension, and remote monitoring or wearable implantable devices including arrhythmia detection. These types of interventions familiarize many patients to telehealth technologies, therefore they may be more willing to engage in them in other settings like oncology [29].

Unlike previous research, we did not find privacy concerns or technical difficulties as major factors associated with reduced telehealth use [30]. Lower odds of older cancer survivors using telehealth may support prior evidence regarding the digital divide; this indicates the need to investigate other potential barriers for older cancer patients and use of telehealth including access to the internet and technology. A study from a primary care setting found that patient portal use was 28%, and patients with the lowest broadband access were less likely to utilize their patient portal [31].

Among the patients who had not utilized telehealth services over the prior 12 months, our study found that 60% of both cancer survivors and the general population were not offered this option.

This may be a result of system-level factors such as medical licensure, payment models and lack of training for implementation of telehealth interventions [32]. However, telehealth regulatory changes during the pandemic led to payment parity and equal reimbursement for telehealth visits as well as elimination of previous licensure requirements [33,34]. Furthermore, patient privacy regulations, particularly certain HIPAA (Health Insurance Portability and Accountability ACT of 1996) requirements, including the use of various video conferencing platforms, were waived during the pandemic [34]. Sequentially, a US Department of Health and Human Services report found a 63-fold increase in telehealth utilization [35]. Hopefully this trend in increased uptake will continue.

Preceding research has established potential benefits of telehealth compared to in person care, including improved quality of life, decreased costs [36,37], and improved access to health care for patients in rural communities [38]. However, research also suggests that telehealth may exacerbate inequities by further increasing the digital divide [39]. To mitigate this risk, specific guidelines and personalized care will need to be implemented. The Journal of the National Cancer Institute has recently published the Framework for Integrating Telehealth Equitably (FITE), which is intended to guide equitable implementation of telehealth [40]. Potential interventions, such as the Telehealth Task Force described by Worster et al [41], have demonstrated that providing tailored digital support can successfully engage older, racially diverse, and socioeconomically disadvantaged cancer patients, thereby helping to reduce the digital divide. A designated task force or telehealth navigators may similarly assist in addressing the needs of older cancer survivors, and those with low health literacy. The lower telehealth utilization observed among employed cancer survivors compared to their unemployed counterparts may reflect scheduling constraints inherent to the working population, such as limited availability during standard clinic hours or difficulty finding a private setting for a telehealth visit during the workday. To address such barriers, the Multinational Association for Supportive Care in Cancer (MASCC)–ASCO standards and practice recommendations for survivorship care emphasize flexible scheduling and the inclusion of both video and audio-only appointment options to increase accessibility across diverse patient populations [42].

### Limitations

Our study does have limitations, as it focuses on data from a snapshot of time in 2022. It should also be noted that this data was collected during the COVID-19 pandemic, which had a large impact on the utilization of telehealth, and it is unclear how telehealth is being used in the post-pandemic setting. The strengths of our study include the inclusion of all cancer types, the large sample size, and the use of a model adjusted for multiple variables—age, sex, race/ethnicity income, education, marital status, BMI, employment status, insurance status, physical activity, diabetes, hypertension, lung disease, heart disease and smoking status—which allowed us to more directly compare the cancer survivor population to the general population.

### Conclusion

As the health care delivery landscape continues to transform, understanding the factors that influence telehealth utilization among cancer survivors is imperative for ensuring equity and access to care, as well as tailoring interventions to meet the unique needs of this population.

Although cancer survivors in our study were significantly more likely than the general population to use telehealth, fewer than half did so. Digital literacy may not be a barrier to telehealth use in cancer survivors as they did not experience more difficulties in their telehealth visits.

The results of our study highlight the need for further efforts to mitigate barriers to telehealth uptake by cancer survivors. Health care providers also need to consider patients’ specific concerns and preferences when promoting telehealth. Future studies are needed to explore and develop personalized approaches to health care delivery, with the goal of optimizing cancer survivors’ outcomes and experiences.

## Abbreviations

FITE: Framework for Integrating Telehealth Equitably
HINTS 6: Health Information National Trends Survey 6
HIPAA: Health Insurance Portability and Accountability ACT
MASCC: Multinational Association for Supportive Care in Cancer

## References

[1] Tonorezos E, Devasia T, Mariotto AB (2024). Prevalence of cancer survivors in the United States. J Natl Cancer Inst.

[2] Nekhlyudov L, Mollica MA, Jacobsen PB, Mayer DK, Shulman LN, Geiger AM (2019). Developing a quality of cancer survivorship care framework: implications for clinical care, research, and policy. J Natl Cancer Inst.

[3] Guy GP, Ekwueme DU, Yabroff KR (2013). Economic burden of cancer survivorship among adults in the United States. J Clin Oncol.

[4] Zheng Z, Jemal A, Han X (2019). Medical financial hardship among cancer survivors in the United States. Cancer.

[5] Paterson C, Bacon R, Dwyer R (2020). The role of telehealth during the COVID-19 pandemic across the interdisciplinary cancer team: implications for practice. Semin Oncol Nurs.

[6] Riera R, Bagattini ÂM, Pacheco RL, Pachito DV, Roitberg F, Ilbawi A (2021). Delays and disruptions in cancer health care due to COVID-19 pandemic: systematic review. JCO Glob Oncol.

[7] Desai A, Gupta R, Advani S (2021). Mortality in hospitalized patients with cancer and coronavirus disease 2019: A systematic review and meta-analysis of cohort studies. Cancer.

[8] Gilroy KJ (2020). Incorporating telemedicine as part of COVID-19 outbreak response systems. Am J Manag Care.

[9] (2018). What is telehealth. NEJM Catal.

[10] Pratt SI, Bartels SJ, Mueser KT (2013). Feasibility and effectiveness of an automated telehealth intervention to improve illness self-management in people with serious psychiatric and medical disorders. Psychiatr Rehabil J.

[11] Garg SK, Parkin CG (2019). The emerging role of telemedicine and mobile health technologies in improving diabetes care. Diabetes Technol Ther.

[12] Wakefield BJ, Holman JE, Ray A (2011). Effectiveness of home telehealth in comorbid diabetes and hypertension: a randomized, controlled trial. Telemed J E Health.

[13] Kroenke K, Krebs EE, Wu J, Yu Z, Chumbler NR, Bair MJ (2014). Telecare collaborative management of chronic pain in primary care: a randomized clinical trial. JAMA.

[14] Larson JL, Rosen AB, Wilson FA (2018). The effect of telehealth interventions on quality of life of cancer patients: a systematic review and meta-analysis. Telemed J E Health.

[15] Ajmera P, Miraj M, Kalra S (2022). Impact of telehealth interventions on physiological and psychological outcomes in breast cancer survivors: a meta-analysis of randomised controlled trials. Front Oncol.

[16] Damholdt MF, Mehlsen M, O’Toole MS, Andreasen RK, Pedersen AD, Zachariae R (2016). Web-based cognitive training for breast cancer survivors with cognitive complaints-a randomized controlled trial. Psychooncology.

[17] Karimi M, Lee E, Couture S (2022). National Survey Trends in Telehealth Use in 2021: Disparities in utilization and audio vs video services. Assistant Secretary for Planning and Evaluation Health and Human Services.

[18] Arthur EK, Pisegna J, Oliveri JM, Aker H, Krok-Schoen JL (2022). Older cancer survivors’ perspectives and use of telehealth in their cancer survivorship care in the United States: a ResearchMatch® sample. J Geriatr Oncol.

[19] Knapp R (2022). Telehealth and equity - AP-NORC. AP-NORC.

[20] (2022). National Cancer Institute. Health Information National Trends Survey 6 (HINTS 6): Annotated English Instrument.

[21] National Cancer Institute. Institutional Review Board (IRB) approvals for the Health Information National Trends Survey (HINTS).

[22] Jewett PI, Vogel RI, Ghebre R (2022). Telehealth in cancer care during COVID-19: disparities by age, race/ethnicity, and residential status. J Cancer Surviv.

[23] Anderson M, Anderson M (2024). 1 Technology use among seniors.

[24] Qian AS, Schiaffino MK, Nalawade V (2022). Disparities in telemedicine during COVID-19. Cancer Med.

[25] Chen LW, Usinger DS, Katz AJ (2023). Telehealth use and perceptions among prostate cancer survivors. Cancer Med.

[26] Picardo E, Baù MG, Anatrone C (2021). Oncophone20 study: Patients’ perception of telemedicine in the COVID‐19 pandemic during follow‐up visits for gynecological and breast cancers. Intl J Gynecology & Obste.

[27] Kjeldsted E, Lindblad KV, Bødtcher H (2021). A population-based survey of patients’ experiences with teleconsultations in cancer care in Denmark during the COVID-19 pandemic. Acta Oncol.

[28] Inglis SC, Clark RA, Dierckx R, Prieto-Merino D, Cleland JG (2015). Structured telephone support or non-invasive telemonitoring for patients with heart failure. Cochrane Libr.

[29] Takahashi EA, Schwamm LH, Adeoye OM (2022). An overview of telehealth in the management of cardiovascular disease: a scientific statement from the American Heart Association. Circulation.

[30] Kato-Lin YC, Thelen ST (2022). Privacy concerns and continued use intention of telemedicine during COVID-19. Telemed J E Health.

[31] Rodriguez JA, Lipsitz SR, Lyles CR, Samal L (2020). Association between patient portal use and broadband access: a national evaluation. J GEN INTERN MED.

[32] Gajarawala SN, Pelkowski JN (2021). Telehealth benefits and barriers. J Nurse Pract.

[33] Shachar C, Engel J, Elwyn G (2020). Implications for telehealth in a postpandemic future. JAMA.

[34] (2021). Admin: COVID-19 telehealth coverage policies - CCHP. CCHP.

[35] (2023). New HHS study shows 63-fold increase in Medicare telehealth utilization during the pandemic. CMS.

[36] Abbott DE, Macke RA, Kurtz J (2018). Financial and temporal advantages of virtual consultation in veterans requiring specialty care. Mil Med.

[37] Beswick DM, Vashi A, Song Y (2016). Consultation via telemedicine and access to operative care for patients with head and neck cancer in a Veterans Health Administration population. Head Neck.

[38] Charlton M, Schlichting J, Chioreso C (2020). Challenges of rural cancer care in the United States. Cancer Network.

[39] Choxi H, VanDerSchaaf H, Li Y, Morgan E (2022). Telehealth and the digital divide: identifying potential care gaps in video visit use. J Med Syst.

[40] Rendle KA, Tan ASL, Spring B (2024). A framework for integrating telehealth equitably across the Cancer Care Continuum. JNCI Monographs.

[41] Worster B, Waldman L, Garber G (2023). Increasing equitable access to telehealth oncology care in the COVID-19 National Emergency: Creation of a telehealth task force. Cancer Med.

[42] Hart NH, Nekhlyudov L, Smith TJ (2024). Survivorship care for people affected by advanced or metastatic cancer: MASCC-ASCO Standards and Practice Recommendations. JCO Oncol Pract.

[43] (2023). Health Information National Trends Survey 6 (HINTS 6): HINTS 6 methodology report. National Cancer Institute.

